# Testicular extracellular matrix/gelatin-based scaffold using gas foaming to support spermatogonial stem cells

**DOI:** 10.22038/ijbms.2025.83885.18150

**Published:** 2025

**Authors:** Maryam Momeni, Mohammad Kazemi Ashtiani, Zahra Bashiri, Zohreh Bagher, Hamidreza Asgari, Morteza Koruji

**Affiliations:** 1 Stem Cell and Regenerative Medicine Research Center, Iran University of Medical Sciences, Tehran, Iran; 2 Department of Anatomy, School of Medicine, Iran University of Medical Sciences, Tehran, Iran; 3 Department of Cell Engineering, Cell Science Research Center, Royan Institute for Stem Cell Biology and Technology, ACECR, Tehran, Iran; 4 Endometrium and Endometriosis Research Center, Hamadan University of Medical Sciences, Hamadan, Iran.; 5 Omid Fertility & Infertility Clinic, Hamedan, Iran; 6 ENT and Head and Neck Research Center and Department, The Five Senses Health Institute, School of Medicine, Iran University of Medical Sciences, Tehran, Iran; 7 Department of Tissue Engineering and Regenerative Medicine, Faculty of Advanced Technologies in Medicine, Iran University of Medical Sciences, Tehran, Iran

**Keywords:** Gas foaming, Pore size, Scaffold, Spermatogonial stem cell Testicular extracellular - matrix

## Abstract

**Objective(s):**

Developing a bioactive testicular scaffold has been proposed as a potential option to preserve male fertility. Using the gas foaming method, this study aimed to fabricate an effective and highly porous scaffold derived from a gelatin-testicular extracellular matrix.

**Materials and Methods:**

Male ram testis decellularization was performed using a combination of NaCl buffer and Triton. Then, evaluations were done for 4′,6-diamidino-2-phenylindole, hematoxylin-eosin staining, and quantitative DNA content. Masson’s trichrome, Alcian blue, and orcein staining were conducted to ensure the maintenance of ECM components post-decellularization. Porous scaffolds were fabricated using gelatin incorporation with various concentrations of extracted ECM via the gas foaming method. The mechanical and structural characteristics of the scaffolds, along with evaluations of cell spreading and penetration depth, were performed. Furthermore, scaffolds were used to culture mouse spermatogonial stem cells to investigate the morphology, viability, and adhesion of the cells on the scaffolds.

**Results:**

Our results showed successful decellularization of testicular tissue, resulting in significant removal of DNA content while preserving ECM major components. The hybrid scaffolds exhibited uniform porous microstructures with a pore size average ranging from 298-330 μm. There were no significant differences in biodegradation and swelling ratios between the scaffolds. The cell penetration index significantly increased in gel-ECM by 5% compared to other groups. Also, ECM5% led to increased cell attachment and viability, proper compressive strength, and a decrease in Young’s module.

**Conclusion:**

Our study suggests that using a combination of testicular ECM and gelatin shows promise in constructing bioartificial testes through the gas-foaming method.

## Introduction

Reduced fertility or even permanent infertility is a common issue among adult males and pediatric cancer survivors who undergo conventional cancer therapy such as surgical operation, radiation therapy, chemotherapy, immunotherapy, and transplantation of stem cells (1, 2). These treatments can damage mature sperm in adults and germ cells in prepubertal boys (3). While numerous approaches have been developed to preserve fertility, most do not apply to prepubertal boys who have not yet begun spermatogenesis and whose testes only contain diploid germ cells, including spermatogonial stem cells (SSCs)(4). Currently, tissue engineering and the use of scaffolds have shown appropriate therapeutic potentials to rectify the issues related to infertility through *in vitro* 3D culture of spermatogonial cells on a scaffold (5-7). Several techniques have been developed for scaffold fabrication, including electrospinning scaffolds, cell-laden hydrogel, and porous scaffolds (8). A scaffold with appropriate porosity can improve cell proliferation, migration, differentiation, and tissue regeneration (9, 10). The parameters of scaffolds, including pore size, interconnectivity, and open porosity, have undoubtedly a crucial role in improving biological applications (11, 12). 

Several publications have reported that modifying the pore size in scaffolds can facilitate oxygen diffusion, nutrient transport, and metabolic waste removal (13, 14). Gas foaming is one of the available methods for manufacturing 3D porous scaffolds, offering a convenient technique to construct highly porous scaffolds (up to 90%) with interconnected structures (15). This method relies on the nucleation and growth of inert gas bubbles, including N_2 _or CO_2_, dispersed throughout a polymer (16). The foaming agent reacts chemically with the polymer, resulting in the formation and release of gas bubbles. Up to now, gas foaming has been widely utilized to create porosity in hydrophobic polymers such as polylactic acid (PLA)(17), Poly Lactic-co-Glycolic Acid (PLGA)(18), and Polycaprolactone (PCL) (19) as well as hydrophilic polymers like alginate (20) and gelatin (21). 

Besides pore diameter and porosity, the composition of scaffolds is also a significant consideration (22, 23). Biological scaffolds containing decellularized testis extracellular matrix (ECM) have emerged as excellent biomedical candidates that mimic the physiological environment of the ECM. These scaffolds contain various collagens, proteoglycans, glycoproteins, elastin, and glycosaminoglycans, essential for testicular cell proliferation and differentiation (24-27). There is no standardized method for decellularization, and various protocols utilizing detergents, enzymes, and physical methods have been explored (28). However, it has been observed that these agents can reduce the amount of ECM components and growth factors (29), alter the ECM architecture (30), and present challenges in removing DNA remnants (31). Nevertheless, the decellularized testicular ECM has been successfully utilized for the proliferation and differentiation of spermatogenic cells, as well as the production of organoids in 3D ECM-containing systems (32), bioprinting technology (33), and ECM-based hydrogels (34). Hybrid ECM-derived scaffolds can be created using various methods. One approach involves using decellularized ECM in a solubilized form (34), combined with a common hydrogel like gelatin derived from collagen hydrolysis (35). Gelatin can be combined with solubilized decellularized ECM to provide structural support to the scaffold (36, 37).

Gelatin is a highly suitable biopolymer for fabricating scaffolds due to its wide availability, biodegradability, inexpensive large-scale production, complete absorbability, and non-immunogenic nature (38-40). Numerous studies have shown that gelatin contains the RGD cell adhesion motif, which plays a significant role in cell binding to the substrate (41-43). These properties make gelatin an excellent choice for constructing foamed scaffolds using the gas-foaming method.

However, the application of the gas foaming method for investigating testicular cell biological functions *in vitro* and the fabrication of foamed testis-derived ECM scaffolds have not been explored yet. Therefore, in the present study, the successful elimination of DNA content from the decellularized ECM extract of ram testicular tissue was first confirmed, along with the preservation of its key factors. Different concentrations of extracted ECM combined with gelatin were used to fabricate hybrid scaffolds using the gas foaming procedure. Subsequently, mechanical tests such as swelling, biodegradability, compressive strength, young modulus, cell adhesion ability, cell survival, cell penetration index, and cytotoxicity *in vitro* were performed to identify the optimal hybrid scaffold composed of gelatin and testicular ECM (Graphical abstract).

**Figure:**
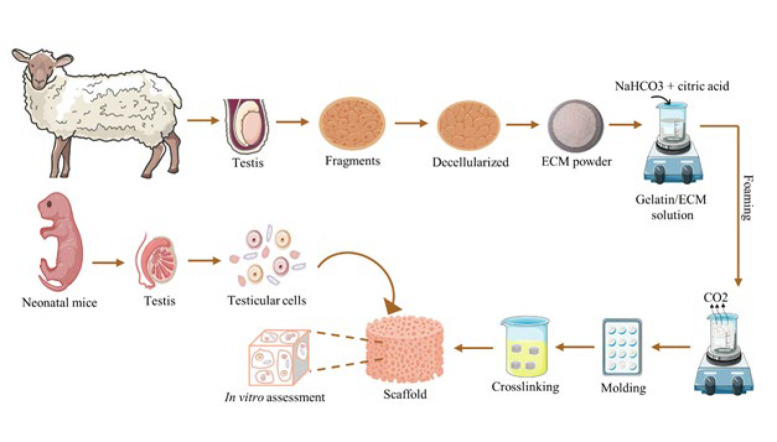
Summary of the study design. Extraction of extracellular matrix (ECM) from ram testis, fabrication of a porous scaffold composed of the extracted ECM and gelatin using gas foaming method, and finally, interaction and evaluation of the testicular cells with the scaffolds

## Materials and Methods

### Animal and ethical considerations

In this study, five healthy one-year-old male rams were slaughtered, and their testes were harvested to obtain testicular ECM. Also, 15 white neonatal male NMRI mice, between 4–6 days old, were included to isolate testicular cells. All mice were kept under standard conditions, including a temperature-controlled room (25±1 ^°^C) with a 12/12-hr light/dark cycle. All animal experiments comply with the ARRIVE guidelines and follow the National Institutes of Health Guide for the Care and Use of Laboratory animals (NIH Publications No. 8023, revised 1978). This work was approved by the Animal Ethics Committee of Iran University of Medical Sciences (Permission no: IR.IUMS.REC.1399.1072).

### Preparation of ram ECM extract

Our previous study (25, 44) successfully decellularized ram testis. Briefly, five one-year-old rams were slaughtered, their testes harvested and placed in a normal saline solution supplemented with gentamicin, and then transported to the laboratory at 4 ^°^C. The testes were decapsulated, sliced into multiple small fragments, and stored at -80 ^°^C until use. 

After dissection of testes, the testicular pieces were homogenized in a Tris-buffered saline (TBS) supplemented by 3.4 M NaCl (Merck, 7647-14-5), 2 mM N-Ethylmaleimide (E3876, Sigma-Aldrich), 50 mM Tris-HCl pH 7.6, and 5 mM EDTA. The homogenized tissues were treated using 1% Triton X-100 (9002-93-1, Sigma-Aldrich) for 15 min. Then, in the subsequent steps, cellular debris was removed by sequential washings and centrifugation steps in TBS buffer for 3 days. The remaining debris was dissolved in a TBS buffer containing 0.15 M NaCl, 4 M urea, and 50 mM Tris for 24 hr and then centrifuged at 15000 rcf for 15 min. The clear and visible supernatant was dialyzed against TBS buffer using a dialysis membrane with a cut-off of 6000-8000 for 24 hr at 4 ^°^C. Finally, the dialyzed solution was lyophilized to obtain a powder, which was stored at -20 ^°^C until use (25, 44)

### Testicular ECM characterization

*Histological evaluation*

The decellularized testicular tissues and the intact tissue (as control) were fixed in Bowen’s solution for 48 hr. Paraffin-embedded blocks of each sample were then sectioned at a thickness of 5 μm. Then, staining with hematoxylin and eosin (H&E), as well as 4′,6-diamidino-2-phenylindole (DAPI)(Sigma, USA), was performed to evaluate the removal of cellular components. The H&E and DAPI stained slides were qualitatively imaged using a light and fluorescent microscope, respectively (Olympus BX51 fluorescent microscope, equipped with a DP72 digital camera, Japan) at a magnification of ×400 (25).

*DNA content quantification*

DNA was extracted from the samples using Trizol^®^ Kit (Kiazist, Iran) following the manufacturer’s protocol (n=3 in each group) to determine and quantify the DNA amount of decellularized and intact tissues. Using the Nanodrop spectrophotometer (Thermo Scientific), the DNA concentration was then quantified at a wavelength of 260 nm and reported as ng/mg of wet tissue weight. Furthermore, gel electrophoresis was performed using a 1% agarose to identify and purify DNA fragments. The samples were loaded into the gel in 1x TBE buffer. Then, GelRed, a nucleic acid stain, was added to the agarose gel to visualize DNA fragments. For each DNA extract, 3 μl was added to 1 μl of loading dye. The size of the DNA fragments was estimated by running a parallel 1 kbp DNA ladder (Fermentas, Invitrogen) on the gel (45).

*Characteristics assessment of the testicular ECM components*

To evaluate the preservation of major ECM components, including collagen, GAG, and elastin, the 5 μm sections of the tissues were stained with Masson’s trichrome, Alcian blue, and Orcein, respectively. To affirm the presence of these factors in the stained slides, ImageJ analysis was conducted on ten randomly selected fields from each group. Additionally, the concentration of collagen and GAG were measured using a collagen Assay Kit (Abcam, ab 222942) and GAG Assay Kit (Abcam, ab 289842). These assays were performed following the manufacturer’s instructions (45). To perform the GAG Assay, 100 mg of ECM was first dissolved in 1 ml of RIPA buffer (cat No. BD9719) and homogenized in a cool place. The mixture was then centrifuged at 4000 rpm for 20 min. The supernatant was separated and stored for subsequent testing. Next, 30 μl of the sample or standard was added to each well of a 96-well plate, and the enzyme solution was diluted to the required concentration and added to each well. Subsequently, 200 μl of GAG reagent was added to each well, and the plate was incubated for 30 sec at room temperature. Finally, the absorbance of the samples was measured at 510-560 nm using an ELISA reader (Biotek-reflex800, USA), and the absorbance results were compared with the standard concentration curve.

### Preparation of ECM solution

The lyophilized ECM powder was mixed with 1 ml Dulbecco’s Modified Eagle medium (DMEM, Gibo) solution in concentrations of 1.5, 3, and 5% to obtain the solubilized ECM. This composition was stirred at 4 ^°^C for 72 hr until the powder was completely dissolved. After dissolution, the resulting solution pH was adjusted to a value of 7.0 by adding 10 M NaOH. Finally, storing the produced ECM solution at 4 ^°^C is suitable for up to two weeks until use.

### Fabrication of scaffolds using the gas foaming method

Using the gas foaming technique, a gelatin scaffold was constructed and characterized to fabricate the porous scaffold. Next, an effective Glutaraldehyde (GTA) concentration as a crosslinker was selected. After that, an ECM solution was prepared, and finally, a porous hybrid scaffold composed of gelatin and ECM different concentrations was produced via a set-upped gas foaming procedure.

*Fabrication of gelatin scaffold*

Using the gas-foaming method, the gelatin scaffold was fabricated following a previously reported procedure (46). For this purpose, gelatin (from pig skin, G1890, Sigma-Aldrich) was added to deionized water to prepare 20% w/v gelatin solution, which was heated to 50 ^°^C and stirred until the gelatin was completely dissolved. To start the foaming process, sodium bicarbonate (NaHCO_3_) was added to the gelatin solution at 0.32 g as a foaming agent. Subsequently, the addition of acetic acid and its reaction with NaHCO_3_ resulted in the formation and release of gaseous carbon dioxide (CO_2_). Rapid production of bubbles and porosity has occurred in the foamed structure. Immediately, the foams were poured into a 12-well plate and frozen at -20 ^°^C for one hour.

After freezing, the foams were rinsed with deionized water to eliminate any remaining unreacted components. Then, the prepared foams were crosslinked with GTA (Merck, 814393) at concentrations of 0.25, 0.5, and 0.75% for three hours. The crosslinked foams were then rinsed in deionized water to eliminate uncross-linked GTA, followed by freezing and lyophilization at -40 ^°^C under a vacuum pressure of 0.250 mbar for 48 hr (46). The stability of the foamed scaffolds with different GTA concentrations was evaluated at 37 ^°^C for one month, and it was found that the foams crosslinked with GTA by 0.25% were completely degraded. Therefore, cell viability was investigated for other foams. For this purpose, spermatogonial stem cells were isolated from mice testis. 

### Spermatogonial cell isolation

Negatatal male mice were used to study the behavior of testicular cells in foamed scaffolds. To obtain the testicular cells, bilateral testes were digested sequentially using an enzymatic digestion solution in two steps (47). In the first step, the tunica albuginea was excised, and the testes were fragmented in DMEM supplemented with an enzymatic digestion solution (0.5 mg/ml collagenase IV, 0.5 mg/ml trypsin, 0.05 mg/ml DNase)(all from Gibco). The resulting mixture of seminiferous tubule fragments and cells was washed through centrifugation (300 rpm for two minutes) to remove interstitial tissue from the testicular pieces. The second enzymatic digestion step followed the same procedure as the first, with testicular fragments incubated in DMEM-containing enzymes. After centrifugation at 32 rcf, testicular cells were collected. To separate germ cells from Sertoli cells, differential plating was performed. Cell suspensions were cultured in DMEM/F12 medium supplemented with 10% fetal bovine serum (FBS, Gibco). After four hours of incubation at 37 ^°^C, Sertoli cells adhered to the bottom of the dish, while the germ cells remained in suspension (47).

*Confirmation of SSCs*

The identity of SSCs was assessed via a flow-cytometry assay using a PLZF marker. Initially, following fixation of 10^5^×1000 cells with 4% paraformaldehyde and 0.02% Triton X100 and rinsing with PBS solution, 10 μl of primary antibody (anti-PLZF antibody, Abcam, ab104854) was added to the cells. After incubating at room temperature for one hour, the cells were rinsed with PBS. Then, 10 μl of secondary antibody (FITC-conjugated) was added, followed by a 30 min incubation at 4 ^°^C. The cells were subsequently rinsed with PBS supplemented with 5% FBS and then fixed in a 4% paraformaldehyde solution. Finally, a flow-cytometry method was employed for the cell analysis. The primary antibody was omitted in the control cells (47).

*Live-dead assay*

SSCs at 1×10^5^ density were cultured on gelatin foams crosslinked with 0.5 and 0.75% GTA for 72 hr to select the optimal GTA concentration. The SSC viability was evaluated using acridine orange/propidium iodide (AO/PI) staining in the live-dead assay. At first, AO (Sigma 9231A) was added to the foams, and the samples were then washed using PBS. Next, the foams were exposed to PI (Sigma 4864P) and were rewashed with PBS. These samples were visualized and imaged using a fluorescence microscope (Olympus). Due to the binding of AO to the nucleic acid, live cells were stained green, and dead cells were stained red by PI due to compromised membranes (48). Ultimately, the optimal GTA concentration was selected to fabricate a hybrid scaffold.

### Fabrication of hybrid scaffold

To prepare the hybrid scaffold, the specified concentrations of ECM (1.5%, 3%, 5%) were mixed into a 20% gelatin solution using a magnetic stirrer. Sodium bicarbonate was added to this composition, followed by acetic acid. This resulted in the fabrication of a foamed structure composed of gelatin and different ECM concentrations using the gas-foaming method. The foamed mixture was poured into a 12-well plate and frozen. Once frozen, the samples were crosslinked by the selected dosage of GTA and then washed overnight. Eventually, the samples were frozen and lyophilized to produce the final hybrid scaffolds.

### Characterization of the prepared scaffolds

Biomechanical and cellular assessments were performed to evaluate the hybrid foamed scaffolds.

*Microstructure analysis and pore size measurement*

The hybrid foamed scaffolds as experimental samples and foamed gelatin scaffolds as control samples were imaged by SEM to visualize the porous structures and define the pore size. For SEM analysis, scaffold specimens were rapidly frozen in liquid nitrogen, freeze-dried for 48 hr, and then affixed to aluminum stubs. Ultimately, all specimens were gold-coated via sputtering and examined by SEM to evaluate the scaffold structure, morphology, porosity, interconnectivity, and pore size. To determine the mean pore size, at least 20 pores from three different areas of a similar sample were measured using Image Analyzer software (ImageJ 1.52v Java 1.8.0-112). The dimensions of the pores were recorded, and the mean pore size was computed. The results were reported as mean values±standard error (46).

*Degradation assay*

The weight loss observation method was employed to calculate the weight loss of the scaffolds over 28 days. Firstly, the weight of the dry scaffolds (M_d_) was measured, and then all of the samples in each group were incubated in the Phosphate-buffered saline (PBS) solution at pH 7.4 and placed at 37 ^°^C. At defined time points (1, 7, 14, and 28 days), the scaffolds were rinsed with distilled water, subjected to freeze-drying, and immediately weighed (M_f_) to record the changes in percentage using the following formula (44).

Weight degraded (%) = [(M_f _- M_d_) / M_d_] × 100 

*Swelling ratio assay*

The scaffolds’ swelling capacity was evaluated by immersing them in PBS (PH 7.4) for specific time points (up to 72 hr) at 37 ^°^C. The initial weight of the dry samples (M_d_ as dry weight) was measured before the immersing process. After the desired time points, the swollen samples (M_s_ as swollen weight) were weighed after gently wiping them off using tissue paper. To calculate the swelling ratio of samples in each group, the following formula (2) was used (44).

Swelling ratio (%) = [(M_s_ - M_d_) / M_d_] × 100 

*Mechanical compression test*

A compressive test was performed on the foamed scaffolds to consider the influence of the foaming procedure on mechanical characteristics. All samples were assessed at a constant of 1 mm S^-1^ using the SANTAM Universal Testing Machine. The top and bottom surfaces of the scaffolds were secured on the compression tester plates to prevent any slipping in the assay. Three parameters of strength, stiffness, and stress/strain ratio were reported in this assay. At first, the resistance of each sample was noted as the peak force, which was used to calculate the maximum stress and report it as the strength of the scaffold. Then, to determine Young’s modulus, the linear segment of the stress-strain curve was used with values reported in kPa to assess the stiffness and mechanical integrity of the scaffolds. Finally, the foamed scaffold’s compressive stress/strain curves were documented (49). 

*Spermatogonial cell cytotoxicity evaluation*

To assess the compatibility of testicular scaffolds, we conducted a cytotoxicity assay using the MTT test (3-(4,5-dimethylthiazol-2-yl)-2,5-diphenyl tetrazolium bromide). This assay allows us to determine the survival of cells in the presence of Hybrid scaffolds composed of different ECM concentrations and gelatin scaffolds. For this experiment, hybrid scaffolds were incubated in culture media at 37 ^°^C for 7 and 14 days. The medium obtained from these samples was subsequently introduced to the cells, with 1 ml of standard culture medium as the control. The cells were seeded in 96-well plates at a density of 1×10^4^ cells per well to adhere and incubate for 24 hr at 37 ^°^C in a 5% CO_2 _incubator. Subsequently, the cells were exposed to the extracted medium for an additional 24 hr. After the incubation period, we added 100 μl of RPMI supplemented with 20 μl of a 1 mM MTT solution to each well. The cells were then incubated for four hours until purple precipitates formed. We aspirated the solution and added DMSO (Thermofisher) to dissolve the purple crystals. Finally, using a microtiter plate reader, we measured the optical density (OD) values of the cells in each well at a wavelength of 540 nm. To calculate the survival rate of the cells, the following formula (3) was used (25). 

Cell survival rate = OD_S_ / OD_C_

OD_S_ denotes the mean optical density of individual samples, while OD_C_ is defined as the mean optical density of the control group.

*Spermatogonial cell distribution throughout scaffold*

To assess *in vitro* cell migration, the scaffolds were initially incubated with DMEM at 37 ^°^C for one hour. Subsequently, 20 µl of fetal bovine serum (FBS, Gibco, 10270106) containing 2×10^5^ mice isolated spermatogonial cells were dropped onto the scaffold. Subsequently, the cell-loaded scaffolds were incubated for 30 min to facilitate cellular penetration into the scaffolds. Following this, 2 ml of complete culture medium (DMEM-F12 containing 10% FBS, 1% penicillin-streptomycin, 1% L-glutamine, 1% NEAA) was added to the plate containing cell-loaded scaffold and incubated for five days. After the incubation period, all scaffolds were fixed in 4% paraformaldehyde, and paraffin blocks were prepared. De-paraffinized sections of slides were then hydrated in a graded alcohol series, and cell nuclei were labeled with DAPI (blue) for 5 min. Finally, the slides were observed using a fluorescent microscope (Olympus, BX51 with Olympus DP72 digital camera). The depth of cell infiltration into the scaffold was determined by counting the blue cell nuclei in the scaffold’s consecutive layers across five random fields within each group. The fold change was determined by comparing the number of cells per scaffold layer and was employed as a cell migration index (50).

*Spermatogonial cell attachment assay*

SEM image analysis was used to assess the quality of cell adhesion to the scaffolds. For this purpose, the cells were seeded on hybrid foamed scaffolds and then incubated at 37 ^°^C. Five days post-cultivation, the cell-scaffold constructs were fixed in 2.5% glutaraldehyde for two hours, dehydrated in a graded ethanol-water series to 100% ethanol, and lyophilized overnight. After that, these constructs were coated with a thin layer of gold, and eventually, the cell adherence to the foamed scaffold was examined using SEM. Next, the cell type was assessed in terms of circularity or expansion by measuring cell size in Image J. The percentage of circular and expanded cells in each group was also calculated.

### Statistical analysis

Data from this study were analyzed using Prism software version 9.5.1. All experiments were conducted independently three times, and values were presented as mean±SD. The student’s t-test was employed to compare two groups, while one-way and two-way analysis of variance (ANOVA) was utilized to compare more than two groups. A *P*-value below 0.05 was considered significant.

## Results

### Characterization of the testicular ECM

*Histological assessment and quantification of DNA content*

**Figure 1:**
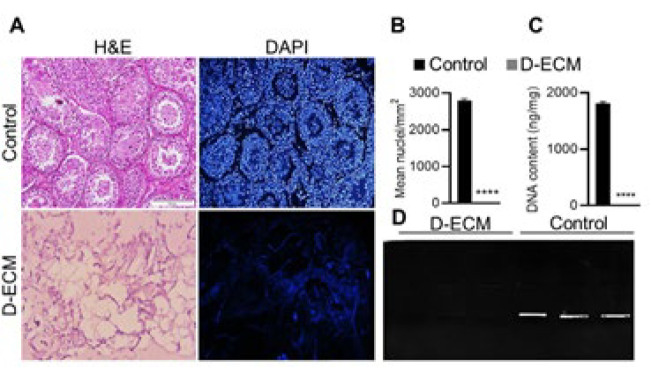
Characterization of the testicular extracted extracellular matrix (ECM) after the decellularization process

In this study, to assess the quality of ECM after the decellularization procedure, histological analysis was done using H&E and DAPI staining. A control sample of native testis tissue was also stained for comparison. Our findings demonstrated a successful decellularization procedure as no cell nuclei were detected in the stained sections of the ECM, in contrast to the control sample (Figure 1A, B). DNA content measurement, another indicator of cellular removal, was carried out to confirm the removal of genetic material from the ECM fragments further. The results determined that a significant proportion of DNA was effectively removed in the decellularized ECM (6.33±0.52) compared to the intact testis tissue (*P*<0.0001). Notably, the standard content of the decellularized ECM in most tissues was reported to be below 50 ng/mg (51), which aligns with our findings (Figure 1C). Additionally, the agarose gel electrophoresis on extracted DNA samples from the decellularized ECM fragments was performed, and no DNA segments were found, unlike the intact tissue (Figure 1D), thus further confirming our previous results.

*Evaluation of the characteristics of the testicular ECM component*

**Figure 2:**
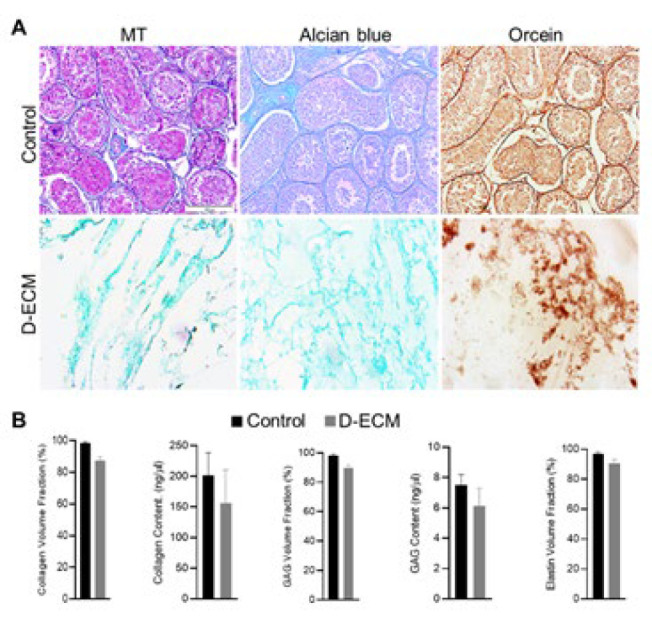
Evaluation of the characteristics of the testicular extracellular matrix (ECM) component

This study evaluated the composition of primary ECM ingredients, including collagen, GAG, and elastin, by specific staining. Masson’s trichrome staining was employed to quantify the collagen amount in the ECM pre- and post-decellularization. Upon evaluation of the stained samples, it is evident that the conserved collagen fibers within the decellularized ECM (Figure 2A, B) closely resembled those found in the intact testis tissue as the control group. This similarity was subsequently verified through quantitative collagen measurements using an ELISA reader (Biotek-refelx800, USA)(Figure 2C).

Alcian blue staining was planned to confirm the preservation of GAGs following successful decellularization. The results demonstrated that GAGs were retained after decellularization, as confirmed by analyzing ten randomly selected sites on each stained slide (Figure 2A). Additionally, by quantitatively evaluating GAG content, no significant difference between ECM pre- and post-decellularization was found (Figure 2B, C).

Furthermore, the presence of elastin fibers, one of the structural elements of ECM, was validated using Orcein staining. The results revealed that the elastin fibers were well preserved following the decellularization procedure (Figure 2A). Upon analyzing the stained samples, we found no significant loss of elastin. All evaluations were performed using the ImageJ software (Figure 2B).

### Characterization of the porous 3D scaffolds

*Characterization of gelatin scaffold*

Crosslinked foams with varying concentrations of GTA were incubated at 37 ^°^C for one month to fabricate a stable porous gelatin scaffold using the gas foaming process. It was found that the scaffold crosslinked with a GTA concentration of 0.25% was disintegrated after a while post-incubation. In contrast, a significant portion of the crosslinked scaffolds with 0.5% and 0.75% GTA concentrations remained intact for the entire month. Subsequently, Live-Dead imaging was performed via SSC culture on gelatin foams to select an optimal GTA concentration. For this purpose, these cells were isolated from testis mice.

*Evaluation of SSCs purity after isolation*

**Figure 3:**
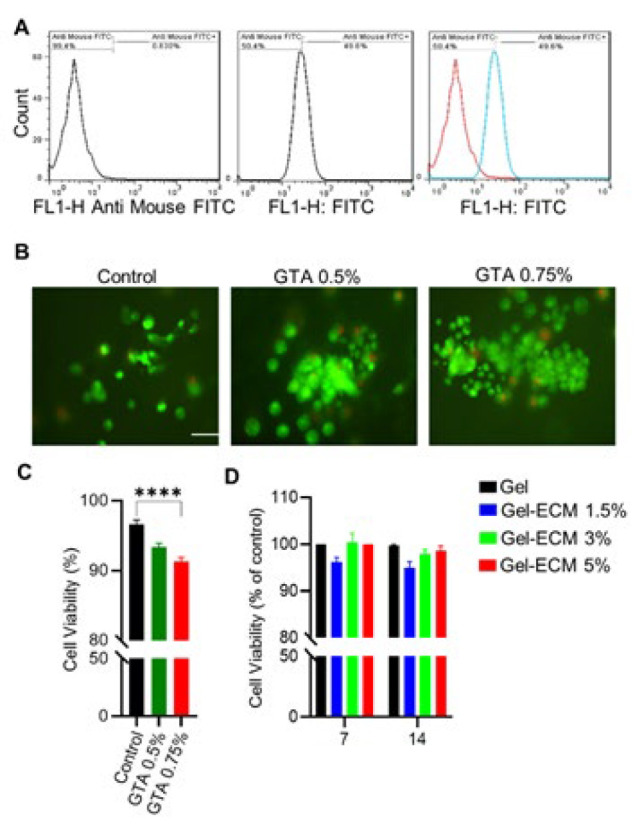
A) Confirmation of spermatogonial stem cell (SSC) purity by flow cytometry for PLZF marker expression. B and C) To choose the optimal GTA concentration as crosslinker, significant decrease of cell viability in the gelatin scaffold crosslinked with 0.75% GTA compared to control via live-dead assay. Mean±SD was expressed and consisted of 3 replications. Scale bar: 200 μm (*P<0.05). D) Evaluation of cellular viability with the MTT method after 7 and 14 days of culture on porous scaffold composed of different concentrations of ECM and gelatin as experiment groups and gelatin scaffold as control group. The results indicated that SSC was more viable in the gel-ECM 5%, but there was no significant difference. Each test was repeated three times (n=3)

To verify the existence of SSCs in isolated cell suspension following enzymatic digestion, the cells were assessed for the PLZF markers through a flow-cytometry assay. The finding indicated that the purity percentage of positive PLZF cells was 49.6% (Figure 3A).

*Live-dead assay*

Live-Dead imaging was performed on scaffolds crosslinked with 0.5% and 0.75% GTA concentration to assess SSC viability. Our findings revealed a marked decrease in cell viability within the scaffolds crosslinked with 0.75% GTA compared to the control group (*P*<0.05). This method used acridine orange and PI dyes to stain live cells (green) and dead cells (red)(Figure 3B, C). Considering the results obtained in the initial phase, a GTA concentration of 0.5% was chosen as the optimal concentration to proceed with the study.

*Spermatogonial cell cytotoxicity evaluation*

In the current study, the gas foaming method was developed and used to fabricate foamed hybrid scaffolds composed of gelatin and 1.5%, 3%, and 5 ECM concentrations. Our pilot study showed that ECM concentrations higher than these values do not lead to the formation of suitable foam, and the foam collapses (data not shown). The MTT test was applied to assess the cytotoxicity of various hybrid scaffolds following exposure to the indirectly obtained extract from the scaffold samples. Our results showed no notable variances in cell survival between the experimental and control groups during the 7- and 14-day post-incubation periods. However, the cell viability in the gel-ECM 5% scaffold was 98.5±1.02, slightly higher than in other samples. Nevertheless, this difference was not statistically significant (*P*=0.05)(Figure 3D).

*Microstructure analysis and pore size measurement of hybrid scaffold*

**Figure 4:**
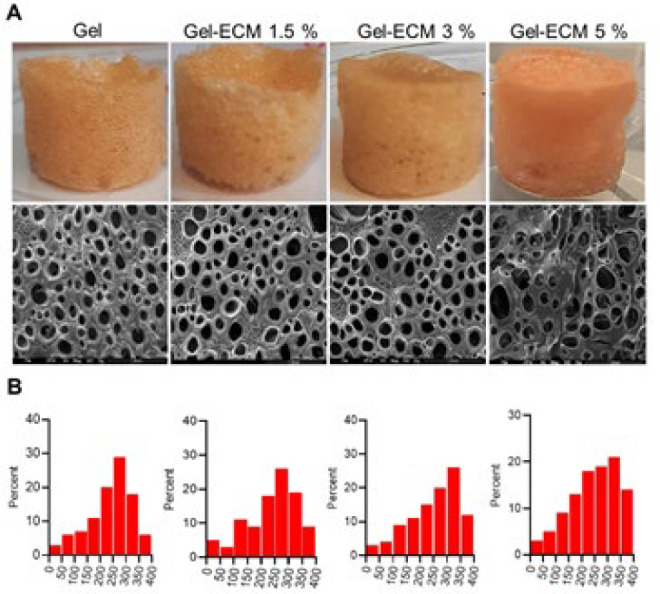
Microstructure evaluation of scaffolds (Gel-different ECM concentration)

SEM analysis was done in this study to understand the scaffolds’ ultrastructure further. Results showed that all foamed 3D porous scaffolds had good porosity and proper interconnectivity between the pores, as depicted in Figure 4A, B. Also, the average pore diameters were measured in each sample; 20 pores were randomly selected and analyzed using ImageJ software. The data indicated that the average pore sizes of the scaffolds were as follows: 296 µm (gel), 298 µm (gel-ECM 1.5%), 321 µm (gel-ECM 3%), and 330 µm (gel-ECM 5%)(Figure 4B). Although the mean pore size increased with higher ECM concentration, no significant difference was observed (*P*=0.05). 

*Degradation assay*

**Figure 5:**
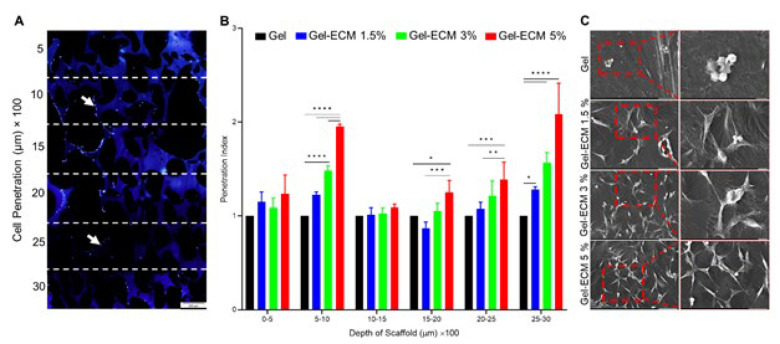
Mechanical properties of hybrid scaffolds (Gel-different ECM concentration)

To consider the *in vitro* degradation of the scaffolds, the weight loss was measured over 30 days. The degradation rates were observed to begin within the first week of incubation. In the experimental groups, the degradation rates were measured as -6.11±3.46, -8.38±1.46, and -7.53±2.93 in the experimental groups, respectively, while the foamed gelatin scaffold had a degradation rate of -10.37±0.64. The scaffold enriched with 1.5% ECM showed significant weight loss compared to the control group at this time point (*P*<0.005). According to the findings, the foamed scaffolds overall remained structurally stable, with less than 26% weight loss after 30 days. Notably, a significant increase in degradation was detected compared to the initial day of the experiment (*P*<0.0001)(Figure 5A).

*Swelling ratio assay*

Regulating nutrients/waste exchange in porous scaffolds relies on possessing an appropriate swelling capacity. In this study, the swelling behavior of porous foamed scaffolds was investigated, and the results indicated that the foamed scaffolds exhibited a swelling capacity ranging from 57% (gelatin) to 78% (gel-ECM 5%) within one hour. This indicates their high porosity and absorption capacity, which was further enhanced by increasing the concentrations of ECM. After 72 hr, the swelling ratio of the scaffolds in the experimental groups continued to increase. The foamed gel-ECM 5% scaffold showed the highest absorption rate (140%), although this difference between all groups was not statistically significant (*P*=0.9)(Figure 5B). The increased swelling capacity observed in foamed scaffolds with higher ECM concentrations aligns with the findings of the degradation assay and suggests a higher susceptibility to hydrolysis reactions.

*Mechanical compression test*

The mechanical features of the foamed scaffolds are illustrated in Figure 5. The ultimate compressive stress (kPa) and strain (%) were determined, and a strain/stress curve was generated to illustrate the correlation between the applied stress (force per unit area) and the resulting strain (deformation)(Figure 5C). This curve helps evaluate the scaffold’s mechanical characteristics, including its strength and stiffness. Our results showed that the Young’s modulus of the samples significantly decreased as the ECM concentration increased (*P*<0.0001)(Figure 5C). 

*Spermatogonial cells distribution throughout scaffolds*

**Figure 6:**
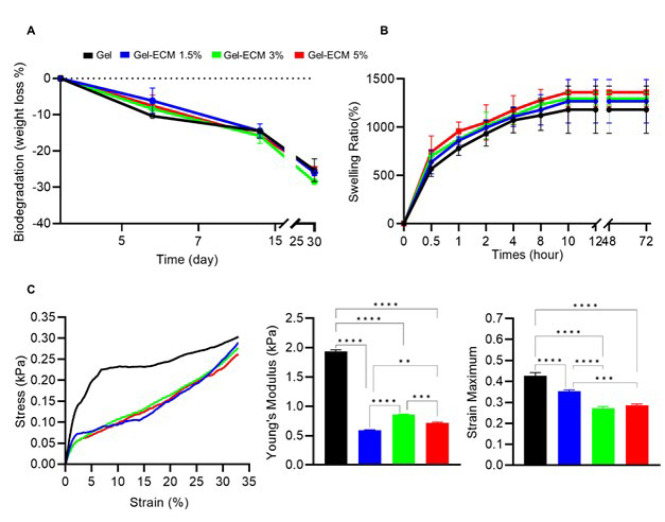
A) DAPI staining of the scaffold on the fifth day to evaluate cellular distribution throughout the scaffold. Scale bar: 200 μm. B) Results showed that cells were significantly migrated up to 3 mm depth of scaffold with higher ECM concentration. Each test was repeated three times (n=3). Data are expressed as mean±SD. (*P<0.05, **P<0.1, ***P<0.001, ****P<0.0001). C) Interaction and adhesion of cells on hybrid scaffolds. Scale bar in left column: 50 μm and in right column: 10 μm

DAPI staining was performed on the fifth day to evaluate cell infiltration within the scaffolds. Our findings revealed a notable penetration of cells into the scaffolds containing 5% ECM compared to the other scaffolds and the foamed gelatin as a control group. The foamed scaffolds constructed using this method exhibited an interconnected structure, allowing cell infiltration in up to 3 mm thick scaffolds. The results indicated that the gel-ECM 5% scaffold exhibited a significant increase in cell penetration (over 2.3 times) compared to other groups (*P*<0.0001)(Figure 6A, B).

*Spermatogonial cell attachment assay*

To evaluate the cell–scaffold interaction, the attachment of cultured SSC cells to hybrid scaffolds was observed using SEM on the fifth day. As depicted in Figure 6C, it was observed that cells were effectively dispersed and adhered to the scaffolds in all groups. Furthermore, there appeared to be increased cell adhesion to the scaffolds with increasing ECM concentrations (Figure 6C).

## Discussion

The main object of this research was to construct a proper 3D scaffold that mimics the microstructure of the testes using decellularized testis ECM. Various scaffolds have been developed by incorporating decellularized testicular ECM in powder, solubilized material (52), or extracted substances (44) into different hydrogels and polymers. However, many of these studies have reported that current decellularization methods can result in damage to the ECM structure and collagen integrity (29), alterations in ECM component content (53), and incomplete removal of cells (54). The present research used a hypertonic NaCl solution to separate DNA from proteins and remove cells from the tissue (52). Triton X-100 was applied to lyse the cells to eliminate the cellular debris (55). Furthermore, urea was used to solubilize the ECM proteins and prepare an extract from the decellularized ECM (56). This study showed that DNA content decreased to less than the standard range (50 ng/mg) post-decellularization, as proved by past studies (44). Additionally, the preservation of collagen, GAG, and elastin was assessed by histological and biochemical evaluations and exhibited that the matrix compounds have been preserved following the decellularization process. However, its amount was reduced compared to the native tissue. Different studies confirmed that our data showed that the ECM proteins were preserved after decellularization (44, 57, 58). These studies indicated that compared with other detergents, Triton X-100 removes cellular content by disrupting lipid-lipid and protein-lipid interactions without affecting protein-protein interactions and better retention of bioactive molecules, which helps preserve the ECM components (59). In our previous study (44) and this project, the decellularization method of testicular ECM with hypertonic NaCl buffer was optimized, and its effectiveness was reported. 

Following the decellularized ECM’s characterization, the study’s next step involved fabricating a gelatin porous and interconnective scaffold using the gas foaming method. To find the optimal concentration of GTA as a crosslinker agent, 0.25%, 0.5%, and 0.75% of the GTA were used to fabricate a porous scaffold. It is well known that the non-toxicity of the crosslinker agent in the scaffolds is crucial for cellular applications. The live-dead assay in our study showed that GTA at higher concentrations than 0.5% v/v reduced cell viability. In a similar study, a 3D porous structure was developed using the gas foaming method, and it has been found that crosslinked gelatin scaffolds with a GTA concentration higher than 0.5% v/v cause decreased cell survival (46,49). Our finding contrasts with some studies reporting cell toxicity at 0.5% v/v concentration of GTA (60), which may be related to the multiple washing of the scaffold in our process, which resulted in the reduction of GTA residues to non-toxic levels. 

One of the main characteristics of a 3D scaffold is an optimal porous structure with an appropriate porosity percentage and interconnected pores, which is crucial for facilitating the diffusion of nutrients, cellular metabolite exchange, and cell-to-cell communication (61). The microstructure of scaffolds containing ECM made by gas foaming methods exhibited larger pores than gelatin. The pore diameter average in the hybrid scaffolds was more than 300 μm, although the gel-ECM 5% samples had the most prominent pores. Overall, pore sizes from 200 to 500 μm are acquired using the gas foaming method, and factors such as temperature, pressure, polymer composition, foaming agent, and crosslinking type and concentration can affect pore sizes (62). Past studies indicated that the average pore dimension in gelatin scaffolds crosslinked with 0.5% v/v GTA fabricated using the gas foaming method was 280 μm. In comparison, this value was 550 µm in structures crosslinked with 1% GTA v/v, although this amount of GTA was toxic (46,49). Also, a porous gelatin scaffold was developed using the gas-foaming method, showing high pore interconnectivity and pore sizes between 100 and 500 μm (63). In the other study, the average pore size in a porous gelatin scaffold prepared with this technique was 250 to 360 μm (21). 

Scaffolds’ degradation and swelling rates are influenced by their components, crosslinking, and fabrication methods (64). Our results indicated that the resulting material showed higher degradation by increasing the ECM concentration in the hybrid scaffold, although almost 74% of the Gel-ECM 5% weight remained 30 days post-incubation. Moreover, water absorption in the scaffold with 5% ECM increased to 140% from the initial day. Our findings showed that the gelatin scaffold demonstrated significantly higher strength and Young’s modulus values than ECM-enriched specimens. According to a previous study, decellularized ECM hydrogels have a lower Young’s modulus than collagen hydrogels (65). Numerous factors impact the mechanical parameters of a fabricated scaffold, such as components, the decellularization process, ECM composition, fabrication method, and scaffold features like pore size (66). It has been reported that Young’s modulus is related to the porosity and pore size of porous platforms. The influence of smaller pore size on higher elastic modulus has been investigated (67). Moreover, a correlation between porosity, pore size, and Young’s modulus has been discovered in past research (61). However, this study did not determine the precise effects of porosity and pore size on Young’s modulus due to the diversity of the tissues studied. Besides, the interaction between collagen in decellularized ECM and the GAG could be responsible for the reduction in Young’s modulus of these structures (68).

An ideal 3D scaffold has other biological properties like non-toxicity and promoting cell adhesion (69). In this study, the toxicity of gelatin-ECM foams to testicular cells was investigated using the indirect MTT method. The testicular cells were exposed to extracts obtained from these scaffolds. Our finding revealed that the extract from ECM-contained foams did not exhibit any toxicity toward cellular growth and viability. Moreover, as the concentration of ECM increased gradually, cells appeared to have longer survival and showed viabilities above 93%. Other studies have confirmed our results by showing that the decellularized ECM is non-cytotoxic when combined with biomaterials (44, 57).

Furthermore, the results of this study indicated that the gel-ECM 5% scaffold supported deeper infiltration and migration of the spermatogonial stem cells. It has been reported that the interplay between two factors, such as the scaffold components and the pore diameter, can affect the ability of cells to penetrate and infiltrate within the scaffold (70). In previous research, a PCL/gelatin scaffold was fabricated via the gas foaming method. It supported human mesenchymal stem cell infiltration up to about 300 μm deep into the scaffold (71). Some studies exhibited that larger pore sizes in porous scaffolds increased the migration speed of human fibrosarcoma and enhanced migration distance for human lymphatic endothelial cells (72). These findings have shown that pore sizes in porous scaffolds can influence proper support for the migration of cells, cell adhesion, and cellular interaction (73). Also, it has been shown that interaction between GAGs as a vital component of the ECM and cell surface receptors mediate biological signals that promote cell migration (74). Moreover, the presence of elastin can influence the ability of cells to migrate and infiltrate (75). These results suggest that the presence of ECM components in the scaffold can induce cell penetration in enriched ECM scaffolds. SEM images showed that with the increase in the concentration of ECM in the scaffold, the number of attached cells increased. It seems that the concentration of ECM components plays an important role in promoting the adhesion of testicular cells (76). Overall, SEM analysis confirmed that the ECM-based scaffolds were suitable for supporting cell attachment.

A) Histological analysis of testicular fragments in control and decellularized ECM in the experiment group. B) DNA content quantification, and C) The average count of 4′,6-diamidino-2-phenylindole (DAPI)-stained nuclei. Mean±SD was expressed and consisted of 3 replications. Scale bar: 200 μm (****P<0.0001). D) Large amounts of DNA were observed in native tissue as control by gel electrophoresis assay

A) Identification of ECM components included collagen, GAGs, and elastin through Masson’s trichrome, Alcian Blue, and Orcein staining, respectively. B) Amount of ECM proteins was quantified by ImageJ analysis, and the amount of collagen and GAGs was measured using an ELISA reader. Data showed all proteins well preserved after decellularization compared to control. All data were expressed as mean/percentage±SD. Scale bar: 200 μm

A) SEM images of scaffold surface. B) Pore size measurement of all scaffolds in different ranges from 50 to 400 µm and calculation of pore size average indicated that the increased pore diameter observed in the gel-ECM 5% group was without significance compared to other groups. Each test was repeated three times (n=3). Scale bar: 1.0 mm

A) Degradation analysis showed that the 5% extracellular matrix (ECM) gel group exhibited less degradation compared to the other groups after 30 days. B) Analysis of the swelling ratio showed that the water absorption ratio in each of the foamed scaffolds increased with the enhancement of the ECM concentration. C) Strain/Stress curve of scaffolds in the compression test. The data showed that including ECM in the scaffolds led to a significant decrease in Young’s modulus. The stiffness comparison of the scaffold based on Young’s modulus values. Each test was repeated three times (n=3). Data are expressed as mean±SD. (**P<0.1, ***P<0.001, ****P<0.0001)

## Conclusion

It was found that the decellularized ECM can be used as a biomaterial to produce scaffolds in combination with natural polymers such as gelatin. In this research, a porous gelatin-testicular ECM scaffold with an interconnected and appropriate pore size was fabricated using the gas foaming method. It was demonstrated that increasing ECM concentration resulted in higher porosity, appropriate pore size, a well-interconnected structure, increased solution absorption, degradability, and deeper migration of spermatogonial cells. Additionally, it was shown that the number of attached cells could be affected by the ECM concentration in scaffolds with larger pores. Based on these findings, we conclude that the porous gel-ECM 5% scaffold has suitable functional applications for the proliferation, growth, and attachment of testicular cells.

## Data Availability

Data will be made available upon request.
